# Genetic insights into the risk of snoring on stroke and ischemic stroke: A single-variable and multivariable Mendelian randomization

**DOI:** 10.3389/fneur.2022.1023748

**Published:** 2022-12-01

**Authors:** Qiang He, Li Ren, Hao Li, Wenjing Wang, Chuanyuan Tao, Lu Ma, Chao You

**Affiliations:** ^1^Department of Neurosurgery, West China Hospital, Sichuan University, Chengdu, China; ^2^Department of Dermatology, West China Hospital, Sichuan University, Chengdu, China; ^3^State Key Laboratory of Proteomics, National Center for Protein Sciences at Beijing, Beijing Institute of Radiation Medicine, Beijing, China; ^4^West China Hospital, Sichuan University, Chengdu, China

**Keywords:** snoring, stroke, ischemic stroke, large artery stroke, cardioembolic stroke, small vessel stroke, Mendelian randomization, causal association

## Abstract

**Background:**

Multiple risk factors of stroke have been identified in previous studies; however, the causal role of snoring in the onset of stroke is less investigated. To clarify the causal association of snoring on stroke and its subtypes, this study is performed.

**Methods:**

The single nucleotide polymorphisms in relation to snoring were retrieved from the UK biobank cohort with 408,317 participants. The data for stroke and its subtypes of European ancestry (67,162 cases and 453,702 controls) were obtained from the MEGASTROKE consortium. In single-variable Mendelian randomization (SVMR) and multivariable MR (MVMR) analyses, inverse variance weighting was used as the primary estimate, complemented with sensitivity analyses more robust to pleiotropy.

**Results:**

Genetically predicted snoring increased the risk of stroke (odds ratio [OR] = 2.69, 95% confidence interval [CI] = 1.19–6.08, *P* = 0.016) and ischemic stroke (IS) (OR = 2.82, 95% CI = 1.23–6.44, *P* = 0.013), but not large artery stroke (LAS) (OR = 3.02, 95% CI = 0.31–29.44, *P* = 0.339), cardioembolic stroke (CES) (OR = 1.51, 95% CI = 0.58–3.92, *P* = 0.395). We provide novel genetic evidence that snoring increases the risk of stroke and IS, but not LAS, CES, and SVS.

**Conclusion:**

Our findings provide novel genetic evidence that snoring increases the risk of stroke and IS, but not LAS, CES, and SVS.

## Introduction

As reported by the Global Burden of Disease Stroke Collaborators, stroke is the second-leading cause of death (1). In 2019, the number of stroke incidents worldwide was 12.2 million, and the related deaths were 6.55 million. Although acute clinical interventions for stroke have advanced substantially since 2015 (2), the disease burden remains significant. Currently, stroke prevention is considered an effective strategy, and 85% of all strokes may be preventable (3). Particularly, the modifiable risk factors, such as smoking, cigarette consumption, total cholesterol, and cigarette consumption, attract growing interest in stroke prevention, as stroke has decreased in incidence by approximately 42% in developed countries within the last 30 years (4). Therefore, identifying and intervening modifiable risk factors may facilitate decreasing the incidence of stroke.

Snoring is the vibration of the upper airway structures causing noise as the air passes in and out during sleep. Habitual snoring is prevalent, and it is estimated that the prevalence is 35–45% in males and 15–28% in females (5). More seriously, the overall incidence of snoring increases with age. Although most patients with obstructive sleep apnea are accompanied by snoring, 20–25% of them with central sleep apnea do not have the symptom of snoring and belong to habitual non-apneic benign snorers (6). Compared with sleep apnea, the potential effect of snoring on stroke has been less studied. In addition, the findings of the association between snoring and stroke remain inconsistent in previous observational studies. For example, snoring in postmenopausal women was associated with stroke (7); however, in a community-based sample over 17 years of follow-up, no significant association was observed between them (8). The discrepancy may be attributed to the study design, limited sample size, and especially the confounders such as snorers accompanying diseases from the cross-sectional or retrospective design in clinics. These biases may impede the yielding of unbiased causal estimates.

To address the inconsistent results in observational design, Mendelian randomization (MR) that can overcome the endogeneity and confounders and then yield causal estimates is selected in this study. MR uses the genetic variants, namely single-nucleotide polymorphisms (SNPs), as instrumental variables (IVs) to examine the causal association between the exposures (i.e., snoring) and outcomes (i.e., stroke) (9). SNPs are assorted randomly in the forming of a zygote during gestation (10). Therefore, MR is similar to the random assortment of interventions in a randomized clinical trial (RCT) and can avoid reverse causation and overcome confounding factors that are typical of non-randomized observational studies (10, 11). At present, no study has been performed to reveal the causal association of snoring on stroke. To clarify the role of snoring on stroke, we performed a single-variable MR (SVMR) and multivariable MR (MVMR) analysis to address the discrepancy and then yield their causal links.

## Methods

### Data sources of snoring, stroke, and its subtypes

The SNPs associated with snoring were obtained from the European ancestry in the UK Biobank (408,000 non-snorers and 152,000 snorers) (12). Snoring was assessed with a question: “Does your partner or a close relative or friend complain about your snoring?”. The corresponding response options were “Yes”, “No”, “Don't know”, or “Prefer not to answer”. The answers “Don't know” or “Prefer not to answer” were removed from the dataset due to the vagueness.

We extracted summary-level datasets of stroke and its subtypes from one meta-analysis by the MEGASTROKE consortium in the European ancestry (13). The dataset of outcomes included stroke (67,162 cases), IS (60,341 cases), LAS (6,688 cases), CES (9,006 cases), and SVS (11,710 cases). The diagnosis of the stroke was based on the World Health Organization (WHO) definition, which was rapidly developing signs of focal (or global) cerebral dysfunction, lasting more than 24 hours or resulting in death with no apparent cause other than that of blood vessel origin. Following the Trial of Org 10,172 in Acute Stroke Treatment (TOAST) criteria (14), IS was subdivided into LAS, CES, and SVS. The detailed information about the study populations, study-specific stroke ascertainment, and subtyping could be accessed through previous publications (13).

### Genetic instrument selection

To retrieve the conditionally independent IVs of snoring, the statistical significance threshold was set at a genome-wide significance level of *P* < 5 × 10^−8^ with linkage disequilibrium (LD) *r*^2^ < 0.01 at a 10,000 kb window size based on 1,000 Genomes European reference panel. We also used the MR-Steiger filtering method to confirm that the SNPs explained more variance in exposure (i.e., snoring) than in outcome (i.e., stroke) (15). When the MR-Steiger test indicated an inverse causality of stroke on snoring, the insignificant SNPs were removed. In our MR-Steiger filtering analysis, all the extracted SNPs passed the test. Besides, the palindromic SNPs were removed.

F-statistics represents the strength of genetic instruments and were calculated using the following formula F-statistics = (Beta/Se) (16, 17). Generally, F-statistic less than 10 was accepted as an indicator of weak IVs, which should be removed. In this step, no SNP was pruned. Additionally, to reduce the heterogeneity and avoid pleiotropy, radial-MR and MR Pleiotropy Residual Sum and Outlier (MR-PRESSO) methods were performed to detect the significant horizontal pleiotropic outliers (18). In these analyses, no significant outliers were detected and then removed, indicating the absence of pleiotropy.

To further verify the absence of possible pleiotropy, we performed a search using an online tool, Phenoscanner (version 2) (http://www.phenoscanner.medschl.cam.ac.uk/), (19) to detect the pleiotropic effects of the selected IVs. We removed 26 SNPs due to their significant links with other diseases and traits (*P* < 5 × 10^−8^), and detailed information was displayed in Supplementary Table S2. Finally, the remaining 17 SNPs (Supplementary Table S1) were selected as the IVs and used to estimate the causal relationship between snoring, stroke, and its subtypes.

### Main statistical analyses

Fixed and random effects inverse variance weighting (IVW) approaches were deemed as the main analyses to test the causal effect of snoring on stroke. When the horizontal pleiotropy is not detected (no violation of the independence assumption) or was balanced, the IVW method can combine the Wald ratios of each SNP to produce an overall unbiased causal estimate of snoring on stroke (20).

A two-sided *P*-value <0.05 was regarded as statistically significant. All statistical analyses were performed using “TwoSampleMR” (20), “MRPRESSO” (18), “mr.raps” (21), and “forestplot” packages in R software (version 3.6.5, Foundation for Statistical Computing, Vienna, Austria).

### Sensitivity analyses

To verify the conformity of the MR results and detect the possible pleiotropy and heterogeneity, we performed four analyses using MR-Egger, MR-PRESSO, Maximum likelihood, and MR robust adjusted profile score (MR-RAPS) methods. In the MR-Egger regression analysis, an intercept term was introduced into the regression model to detect the directional pleiotropy. Even if all the instruments were invalid, MR-Egger could yield valid causal effect estimates (22). MR-PRESSO was used to detect horizontal pleiotropy, correct the significant outliers, and further produce a more robust estimate (18). Maximum likelihood method could obtain a causal effect by the direct maximization of the likelihood, and assume a linear relationship between the exposure (i.e., snoring) and outcome (i.e., stroke). After modeling by MR-RAPS, the robust results could be produced under the assumption of the normal distribution of pleiotropic effects. Even when the weak IVs and systematic and idiosyncratic pleiotropy existed, the findings from MR-RAPS were robust.

The Cochran's Q test was applied to test the heterogeneity across all instrumental SNPs. In addition, the “leave-one-out” sensitivity analysis was used to evaluate whether the snoring-stroke causal links were driven by influential SNPs, otherwise indicating the robustness of the casual estimation.

The statistical power to detect the difference was calculated using an online tool (https://shiny.cnsgenomics.com/mRnd/). When the threshold of type I error rate was 0.05, the statistical power of snoring on stroke was 100%. In addition, we calculated the bias and overlap in the website “https://sb452.shinyapps.io/overlap/”. When the threshold of type I error rate was 0.05 and the overlap proportions were 100%, the value of the bias was 0.056. This finding indicated that our results were stable the statistical power was ample and the bias from sample overlap seemed to be minimal in this study.

### Multivariable MR of snoring on stroke and its subtypes

To investigate the direct causal effect of snoring on stroke and its subtypes, we performed MVMR analysis (23). MVMR can detect causal effects of multiple risk factors on stroke jointly, and further obtain the independent association of each risk exposure with the outcome (20, 24). In previous studies, snoring could be influenced by other heritable lifestyle factors such as smoking and alcohol drinks (25–27). Therefore, the potential confounders in the MVMR analyses in our study included smoking, alcoholic consumption, low-density lipoprotein (LDL), total cholesterol (TC), and body mass index (BMI). The SNPs in MVMR analyses were the overlapping SNPs between snoring and the confounders.

## Results

### Causal effect estimates of snoring on stroke in SVMR

**Figure 1:**
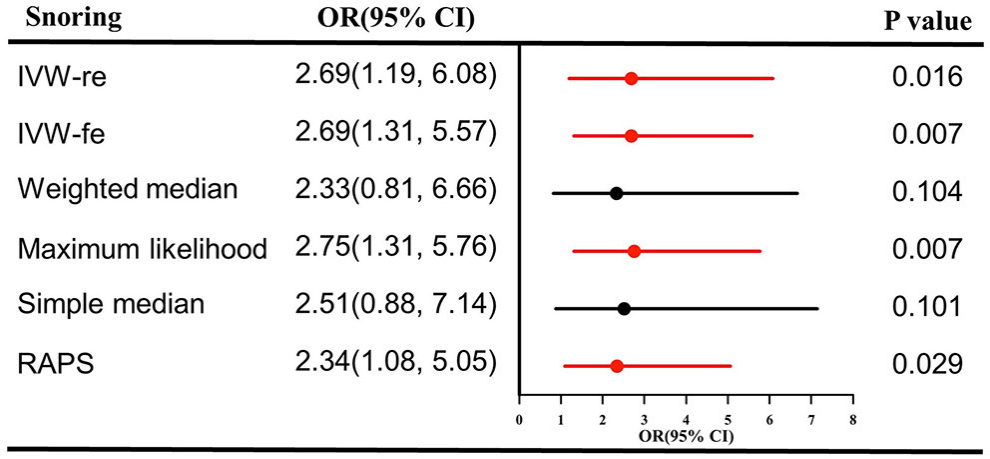
Causal effect estimates of snoring on stroke in SVMR. OR, odds ratio; CI, confidence interval; IVW, inverse variance weighted method; RAPS, robust adjusted profile score; MR, Mendelian randomization; SVMR, single-variable Mendelian randomization.

**Figure 2:**
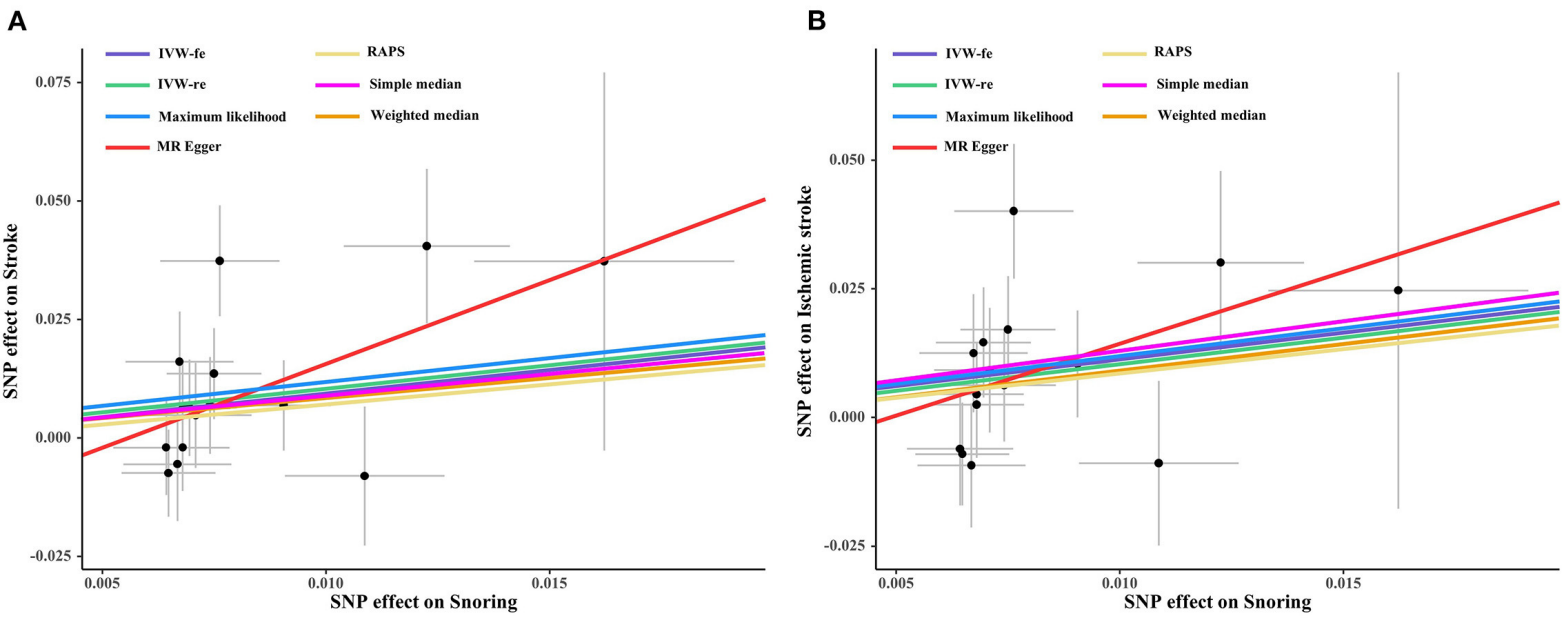
Scatter plot of the effect size of each SNP on snoring, stroke **(A)** and IS **(B)** in SVMR. SNP, single nucleotide polymorphism; IS, ischemic stroke; IVW, inverse variance weighted method; MR, Mendelian randomization.

As shown in Figure 1, genetically predicted snoring causally lead to a 2.69-fold increase in stroke risk [95% confidence interval (CI)] = 1.19–6.08, *P* = 0.016 for the IVW-re estimator; 95% CI = 1.31–5.57, *P* = 0.007 for the IVW-fe estimator. The scatter plot in Figure 2A showed that with the increase of IVs' effect on snoring, the SNPs' effect on stroke increased. In sensitivity analyses, the causal association between snoring and stroke still existed (Maximum likelihood method: OR = 2.75, 95% CI = 1.31–5.76, *P* = 0.007; MR-RAPS: OR = 2.34 95% CI = 1.08–5.05, *P* = 0.029; Figure 1).

In sensitivity analysis, there was no signs of pleiotropy (MR-Egger: intercept term = −0.019; *P* = 0.251, Table 1; MR-PRESSO global test: *P* = 0.330). The heterogeneity was not observed according to the results of Cochran's *Q* statistics (*P* > 0.330) (Table 1) and the funnel plot (Supplementary Figure S1), indicating that no significant outliers were detected. Furthermore, the results from the leave-one-out method revealed that the positive association remained robust after leaving any single SNP out in turn (Supplementary Figure S2). This indicated that no influential SNPs were found. The forest plot visualizing the effect estimate of each SNP on stroke was displayed in Supplementary Figure S3.

**Table 1:** MR estimates from each method of the causal effect of snoring on stroke and its subtypes.

**Traits**	**MR methods**	**OR**	**95% CI**	***P***	**Cochran's Q statistic**	**Heterogeneity *P-*value**	**MR-Egger intercept**	**Intercept *P-*value**
Stroke	MR-Egger	34.48	0.50–2,373.70	0.125	15.861	0.256	−0.019	0.251
	IVW-re	2.69	1.19–6.08	0.016	17.623	0.224	-	-
	Maximum likelihood method	2.75	1.31–5.76	0.007	17.441	0.233	-	
IS	MR-Egger	16.326	0.15–1,366.77	0.238	14.781	0.321	−0.013	0.442
	IVW-re	2.820	1.23–6.44	0.013	15.493	0.345	-	-
	Maximum likelihood method	2.936	1.31–6.54	0.008	15.363	0.353	-	-
LAS	MR-Egger	0.163	5.10e-07–52,318.92	0.783	18.672	0.133	0.022	0.653
	IVW-re	3.027	0.31–29.44	0.339	18.975	0.165	-	-
	Maximum likelihood method	3.145	0.42–23.01	0.259	18.948	0.166	-	-
CES	MR-Egger	5.993	0.01–21,971.55	0.675	5.462	0.963	−0.010	0.743
	IVW-re	1.512	0.58–3.92	0.395	5.574	0.976	-	-
	Maximum likelihood method	1.528	0.33–6.98	0.584	5.571	0.976	-	-
SVS	MR-Egger	4.449	0.01–74,117.93	0.768	9.302	0.749	0.037	0.898
	IVW-re	2.360	0.53–10.45	0.258	9.319	0.810	-	-
	Maximum likelihood method	2.439	0.38–15.35	0.342	9.308	0.810	-	-

*MR*, Mendelian randomization; *OR*, odds ratio; *CI*, confidence interval; *IS*, ischemic stroke; *LAS*, large artery stroke; *CES*, cardioembolic stroke; *SVS*, small vessel stroke; *IVW*, inverse variance weighted.

### Causal effect estimates of snoring on IS in SVMR

**Figure 3:**
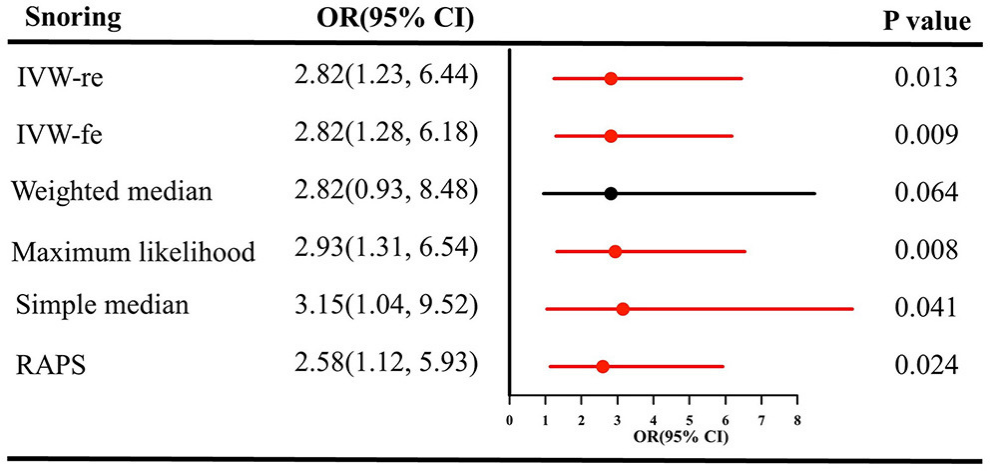
Causal estimates of snoring on IS in SVMR. OR, odds ratio; CI, confidence interval; IVW, inverse variance weighted method; IS, ischemic stroke; RAPS, robust adjusted profile score; MR, Mendelian randomization; SVMR, single-variable Mendelian randomization.

Likewise, snoring increased the risk of IS **[(**OR = 2.82, 95% CI = 1.23–6.44, *P* = 0.013 for the IVW-re estimator; OR = 2.82, 95% CI = 1.28–6.18, *P* = 0.009 for the IVW-fe estimator); Figure 3]. The results were similar in maximum likelihood method (OR = 2.93, 95% CI = 1.31–6.54, *P* = 0.008); simple median (OR = 3.15, 95% CI = 1.04–9.52, *P* = 0.041), and MR-RAPS (OR = 2.58, 95% CI = 1.12–5.93, *P* = 0.024). As shown in Figure 2B, the risk of IS increased as the IVs' effect on snoring increased.

No pleiotropy was identified using MR-Egger [(intercept term = −0.013; *P* = 0.442), Table 1] and MR-PRESSO methods (all *P* = 0.473) in sensitivity analyses. The results of Cochran's *Q* test revealed the absence of heterogeneity [(*P* > 0.05), Table 1]. The heterogeneity results visualized in the funnel plot were presented in Supplementary Figure S4. Additionally, no influential IVs were identified in the leave-one-out analysis when excluding any one of SNP in turn (Supplementary Figure S5). The estimates from each IV were presented in Supplementary Figure S6.

### Causal effect estimates of snoring on LAS, CES, and SVS in SVMR

As shown in Supplementary Figures S7–S9, snoring was not causally associated with LAS, CES, and SVS (all *P* > 0.05). The effect of snoring on LAS, CES, and SVS visualizing in the scatter plot revealed that the snoring did not increase their risk (Supplementary Figures S1–S12). There were no signs of heterogeneity according to the results of Cochran's *Q* tests [(*P* > 0.05); Table 1, Supplementary Figures S13–S15]. The results in the leave-one-out analysis for LAS, CES, and SVS remained consistent when excluding any one SNP at a time (Supplementary Figures S16–S18). The casual estimates from each IV on LAS, CES, and SVS were presented in Supplementary Figures S19–S21.

### Causal effect estimates of snoring on stroke and its subtypes in MVMR

**Figure 4:**
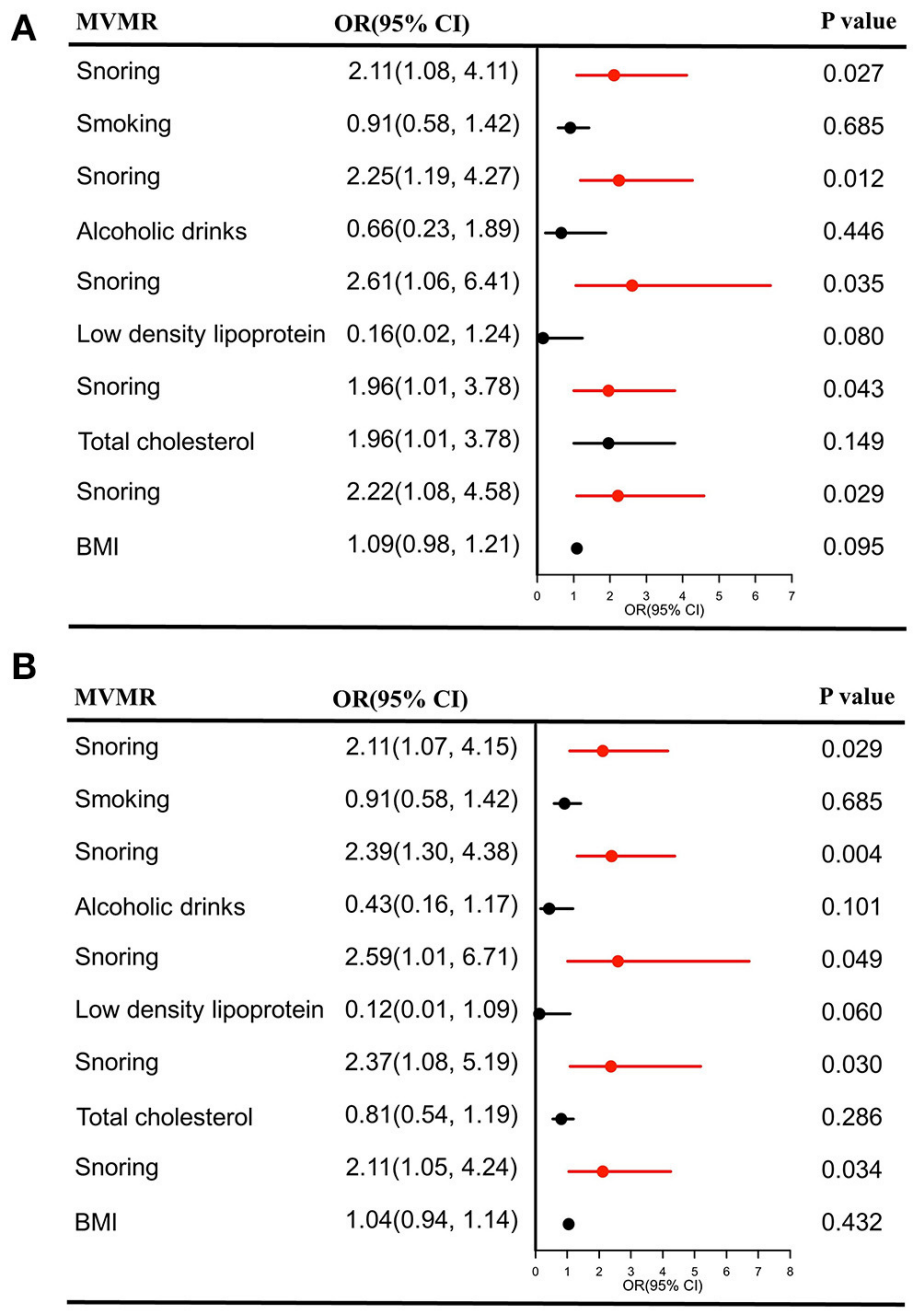
Causal estimates of snoring on stroke **(A)** and IS **(B)** in MVMR. OR, odds ratio; CI, confidence interval; IS, Ishemic stroke; MVMR, multivariable Mendelian randomization; BMI, body mass index.

As shown in Figure 4A, the casual effects of snoring on stroke remained unchanged after adjusting for smoking (OR = 2.11, 95% CI = 1.08–4.11, *P* = 0.027); alcoholic drinks (OR = 2.25, 95% CI = 1.19–4.27, *P* = 0.012), LDL (OR = 2.61, 95% CI = 1.06–6.41, *P* = 0.035), TC (OR = 1.96, 95% CI = 1.01–3.78, *P* = 0.043), and BMI (OR = 2.22, 95% CI = 1.08–4.58, *P* = 0.029), respectively. The positive association still existed for IS in MVMR (smoking: OR = 2.11, 95% CI = 1.07–4.15, *P* = 0.029; alcoholic drinks: OR = 2.39, 95% CI = 1.30–4.38, *P* = 0.004; LDL: OR = 2.59, 95% CI = 1.01–6.71, *P* = 0.049; TC: OR = 2.37, 95% CI = 1.08–5.19, *P* = 0.031; BMI: OR = 2.11, 95% CI = 1.05–4.24, *P* = 0.034), (Figure 4B).

No significant casual association was detected for LAS, CES, and SVS after adjusting the confounders [(all *P* > 0.05), Supplementary Figures S22–S24]. SVMR analysis indicated no causal effects of snoring on these subtypes. Therefore, our analysis provided evidence that snoring could not increase their risk.

## Discussion

Snoring is a health problem, and studies in adults and children suggest that the frequency of snoring can predicts symptoms and poorer behavioral and cognitive outcomes (28, 29). Using GWAS summary data, our study identifies the causal association between snoring, stroke, and IS. In addition, no causal association is observed between snoring, LAS, CES, and SVS.

Some previous large population-based cohort studies and meta-analyses may support our conclusion. For instance, during a median follow-up of 9.6 years in a China Kadoorie Biobank (CKB) study of 489,583 participants, habitual snoring increased the risk of IS (hazard ratio = 1.12) among participants aged <50 years (30), while such associations did not exist among individuals in adults aged over 50 years. However, obvious limitations should be mentioned in their study. Firstly, the snoring status of participants was available only at baseline for most CKB participants. However, the condition of these people remained unclear during other period. Moreover, some participants who were unaware of their snoring status were assigned as non-snorers. Therefore, these findings about the association between stroke risk and snoring could not be verified whether habitual snoring was associated with stroke risk and required further research. Recently, a result of a meta-analysis including 3,598 stroke patients and 145,901 participants without stroke, suggested that snoring was associated with a significantly increased risk of stroke (relative risk 1.46; 95% CI, 1.29–1.63; *P*< 0.001) (31). A similar meta-analysis conducted by Min Li et al. reported that snoring had a modest but statistically significant positive association with the risk of stroke (32). However, the studies included in the meta-analysis varied in study design, population, adjustment for confounders, and different diagnostic methods for exposure and outcome, which reduced the reliability of their results. Performing RCT about the association between snoring and stroke seems difficult in practice. Our MR analysis provides novel evidence to overcome these confounders and discloses the casual association of snoring on stroke.

The insignificant results between snoring and stroke are also reported elsewhere. In the Busselton Health Study, no significant association was identified between snoring and stroke during a follow-up of 17 years (8). However, this study had limited sample size (360 participants), reducing the reliability of the conclusion. Besides, a population-based cohort in the Jackson Heart Study with 4,495 participants (787 snorers and 3,708 non-snorers) found that self-reported habitual snoring was not associated with incident stroke (33). Yet, subjects in the study were African Americans, who had the greatest level of difficulty in recalling snoring, especially males (34). Furthermore, some individuals among non-whites remained uncertain whether they had snoring status, or reported snoring inaccurately. These would lead to the low prevalence of snoring and further make an opposite conclusion. The causal association of snoring on stroke is identified in our study. This causal report addresses the discrepancy in previous observational studies and may support the clinical decision about identifying snoring for preventing stroke.

The exact mechanisms linking snoring to stroke remain unclear. One possible explanation may be the hypoxia and the vibration of the upper airway structures during sleep according to the definition of snoring (35, 36). Obstruction or stenosis of the upper airway could lead to hypoxia and further result in the chronic activation of the sympathetic system, oxidative stress, and inflammation, all of which were involved in the pathological process of hypertension and atherosclerosis (37). In western countries, some studies suggested that patients with habitual snoring had a higher risk of hypertension than their non-habitual snoring counterparts (38, 39). Moreover, preload and afterload of the heart increased during snoring due to large swing in pleural pressure, and snoring-related energy could be transmitted to the caroid artery and further induce the process of atherosclerosis or the disruption of atherosclerotic plaques (40, 41). In addition, the high energy generated by vibration during snoring could be transmitted to the proximal tissues including the carotid artery, which might cause cascade effects of numerous cells in the arterial wall and lead to alteration in vascular structure and function (41, 42).

In the analysis of stroke subtypes, association between snoring and IS was observed, while the causal association does not exist between snoring and LAS, CES, and SVS. These results indicate that biological heterogeneity of the genetic effect of snoring on different IS subtypes may exist, and different subtypes may have distinct pathological mechanisms.

There are several strengths in our MR study. Firstly, we clarify the causal association between snoring and stroke using the MR method, which overcomes the underlying impact of confounding factors and yields causal inferences. Moreover, this study is the first study to directly identify the causal effects of snoring on stroke and its subtypes, which can assist doctors in the clinical decision. The association between other sleep disorders, such as insomnia and sleep duration, have been clarified in MR analyses. In our study, we found the casual association between the snoring and stroke and IS. However, there are some shortcomings in our study. The main limitation is that the datasets originate from the European population, which limits the generalization of the conclusion. Furthermore, only self-reported information on snoring was available. In addition, the case of stroke subtypes was relatively small. Therefore, we should evaluate the conclusion with caution. In future studies, replication in other ancestries, more rigorous clinical study design, and large studies with more samples should be performed to verify the conclusions.

## Conclusions

In conclusion, our study supports the causal association between snoring, stroke, and IS. In clinical settings, snoring should be noted by doctors, and interventions targeting snoring should be considered.

## Data availability statement

The original contributions presented in the study are included in the article/Supplementary material, further inquiries can be directed to the corresponding authors.

## Ethics statement

Ethical review and approval was not required for the study on human participants in accordance with the local legislation and institutional requirements. The patients/participants provided their written informed consent to participate in this study.

## Author contributions

QH and LR analyzed and interpreted the data and wrote the manuscript. HL, WW, and QH analyzed the data. CT, LM, and CY designed the study. All authors contributed to the article and approved the submitted version.

## Funding

This study was supported by the 1·3·5 project for disciplines of excellence—Clinical Research Incubation Project, West China Hospital, Sichuan University (2018HXFH010).

## Conflict of interest

The authors declare that the research was conducted in the absence of any commercial or financial relationships that could be construed as a potential conflict of interest.

## Publisher's note

All claims expressed in this article are solely those of the authors and do not necessarily represent those of their affiliated organizations, or those of the publisher, the editors and the reviewers. Any product that may be evaluated in this article, or claim that may be made by its manufacturer, is not guaranteed or endorsed by the publisher.
