# Supplementary material for: Genetic insights into the risk of snoring on stroke and ischemic stroke: A single-variable and multivariable Mendelian randomization
Source: Front Neurol. 2022 Dec 1;13:1023748. doi: 10.3389/fneur.2022.1023748 (PMC9754687; doi:10.3389/fneur.2022.1023748)
Supplement: Supplementary file 1 [file Data_Sheet_1.docx]

**Table S1** SNPs used as genetic instruments in the Mendelian randomization analyses

| SNP | Chr | Effect allele | Other allele | Beta. exposure | P | Se |
| --- | --- | --- | --- | --- | --- | --- |
| rs12119849 | 1 | A | G | 0.012256 | 4.10E-11 | 0.001860 |
| rs2115855 | 2 | G | T | 0.006430 | 3.60E-08 | 0.001186 |
| rs1374895 | 3 | T | C | -0.006480 | 4.50E-10 | 0.001051 |
| rs17060460 | 6 | G | A | 0.007091 | 1.40E-08 | 0.001247 |
| rs947612 | 6 | A | G | -0.006730 | 1.50E-08 | 0.001210 |
| rs7829639 | 8 | G | A | 0.007413 | 1.40E-10 | 0.001155 |
| rs11018488 | 11 | T | A | -0.006780 | 5.30E-10 | 0.001099 |
| rs12429765 | 13 | G | A | -0.006800 | 6.20E-11 | 0.001051 |
| rs592333 | 13 | G | A | 0.009058 | 1.00E-17 | 0.001051 |
| rs9583546 | 13 | C | G | 0.005868 | 4.00E-08 | 0.001080 |
| rs2664299 | 14 | C | T | -0.007500 | 1.10E-12 | 0.001061 |
| rs57292959 | 16 | T | G | 0.006950 | 5.10E-11 | 0.001059 |
| rs180110 | 17 | A | G | 0.006801 | 2.10E-10 | 0.001060 |
| rs4987719 | 18 | T | C | 0.016225 | 3.50E-08 | 0.002906 |
| rs8108822 | 19 | T | C | -0.010870 | 6.20E-10 | 0.001783 |
| rs4815897 | 20 | G | A | 0.007629 | 1.20E-08 | 0.001333 |
| rs6099273 | 20 | T | C | 0.006682 | 2.60E-08 | 0.001206 |

SNP: [single](javascript:;) [nucleotide](javascript:;) [polymorphism](javascript:;); SE: standard error.

**Table S2** The removed SNPs in the PhenoScanner (version 2) search

| SNP | *P* value | Diseases and traits | PMID |
| --- | --- | --- | --- |
| rs1416685 | 1.95e-11 | Peak expiratory flow | UKBB |
| rs33998002 | 1.12e-08 | Basophil percentage of granulocytes | 27863252 |
| rs75144690 | 1.44e-08 | Red cell distribution width | 27863252 |
| rs61597598 | 3.94e-13 | Forced expiratory volume in 1-second | UKBB |
| rs1609721 | 5.00e-16 | Body mass index | UKBB |
| rs34811474 | 5.30e-27 | Body mass index | UKBB |
| rs13156484 | 3.24e-39 | Whole body fat | UKBB |
| rs2307111 | 6.35e-32 | Body mass index | UKBB |
| rs4976269 | 3.45e-08 | Forced vital capacity, best measure | UKBB |
| rs2207944 | 2.29e-09 | Nap during day | UKBB |
| rs17151229 | 4.06e-10 | Worrier or anxious feelings | UKBB |
| rs13251292 | 3.55e-18 | Heel bone mineral density | UKBB |
| rs4744369 | 8.09e-15 | Height | UKBB |
| rs725861 | 4.42e-27 | Asthma | UKBB |
| rs2049045 | 2.70e-38 | Body mass index | UKBB |
| rs10878269 | 8.14e-14 | Height | UKBB |
| rs2277339 | 3.50e-18 | Height | 28146470 |
| rs1985704 | 2.06e-33 | Height | UKBB |
| rs1108431 | 1.13e-13 | Hip circumference | UKBB |
| rs17680229 | 1.71e-09 | Hand grip strength right | UKBB |
| rs227731 | 3.10e-18 | Height | 25282103 |
| rs55938136 | 8.12e-34 | Nap during day | UKBB |
| rs57222984 | 1.11e-30 | Nap during day | UKBB |
| rs8069947 | 6.73e-12 | Systolic blood pressure | UKBB |
| rs9900496 | 2.79e-08 | Testosterone | 28887542 |
| rs6054427 | 2.18e-87 | Height | UKBB |


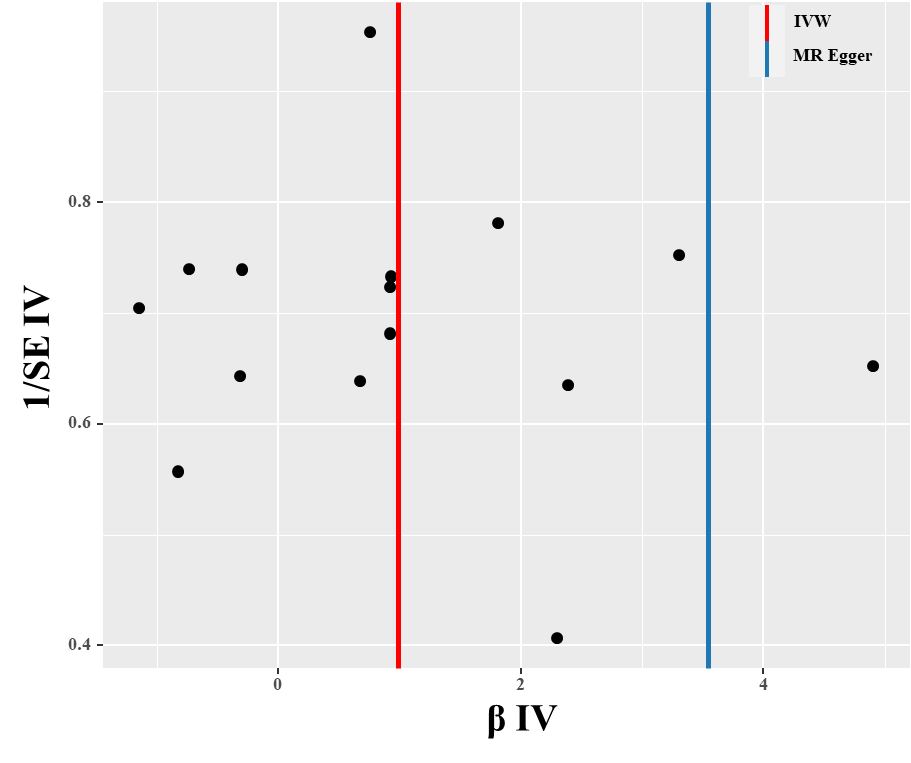


**Figure S1** The funnel plot between genetically predicted snoring and stroke in SVMR. IVW, Inverse variance weighted method; SVMR, single-variable mendelian randomization. IV, instrumental variable; SE, standard error; MR, mendelian randomization.


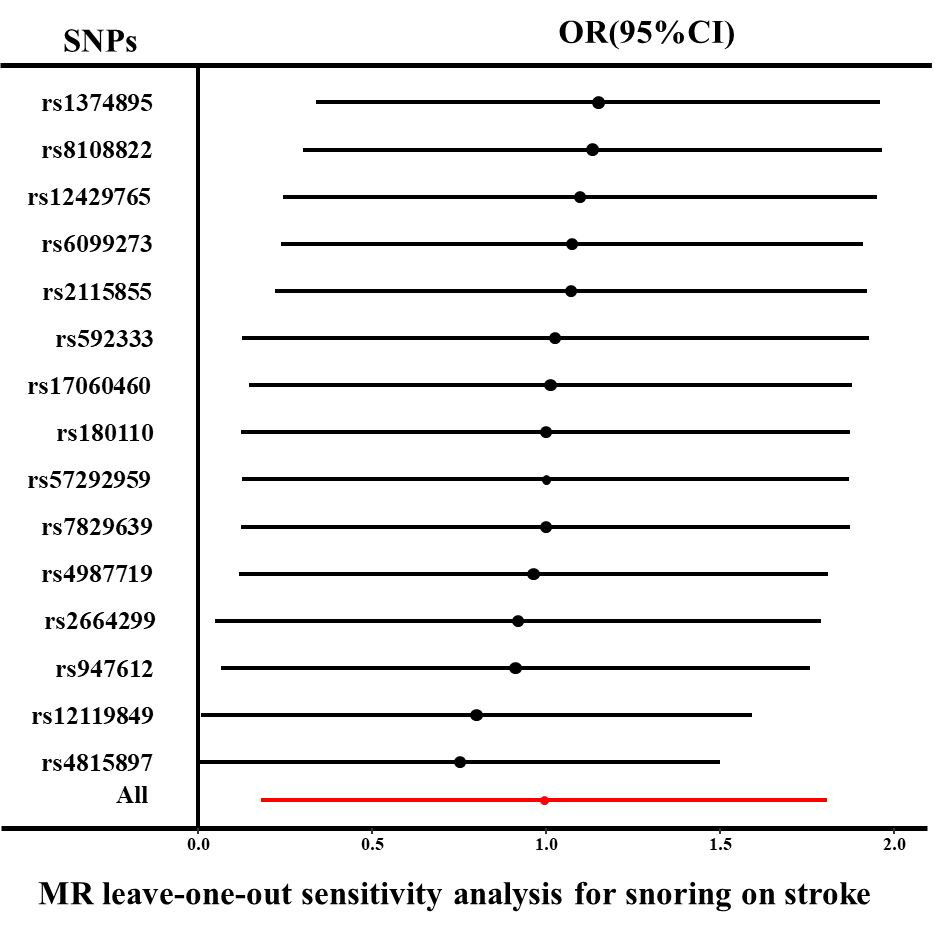


**Figure S2** The leave-one-out sensitivity analyses of snoring and stroke. SNP, single nucleotide polymorphism; OR, odds ratio; CI, confidence interval; MR, mendelian randomization.


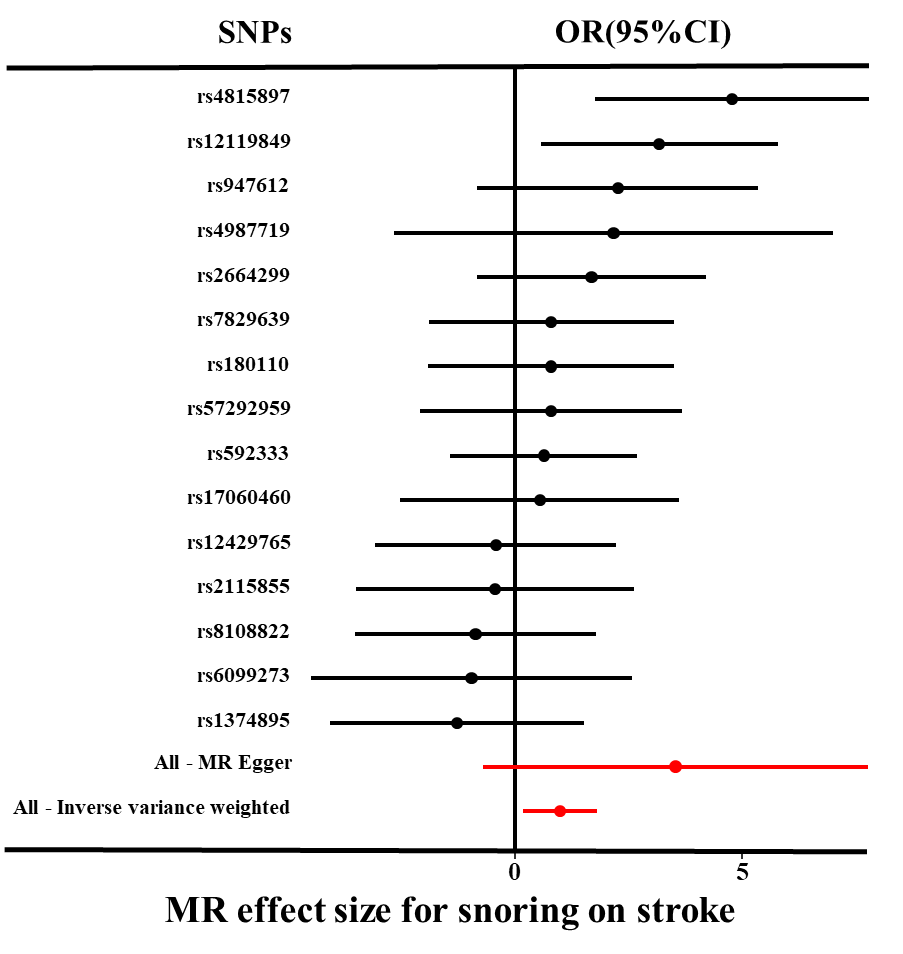


**Figure S3** The results of MR analyses of causal associations between each snoring SNP and stroke. SNP, single nucleotide polymorphism; OR, odds ratio; CI, confidence interval; MR, mendelian randomization.


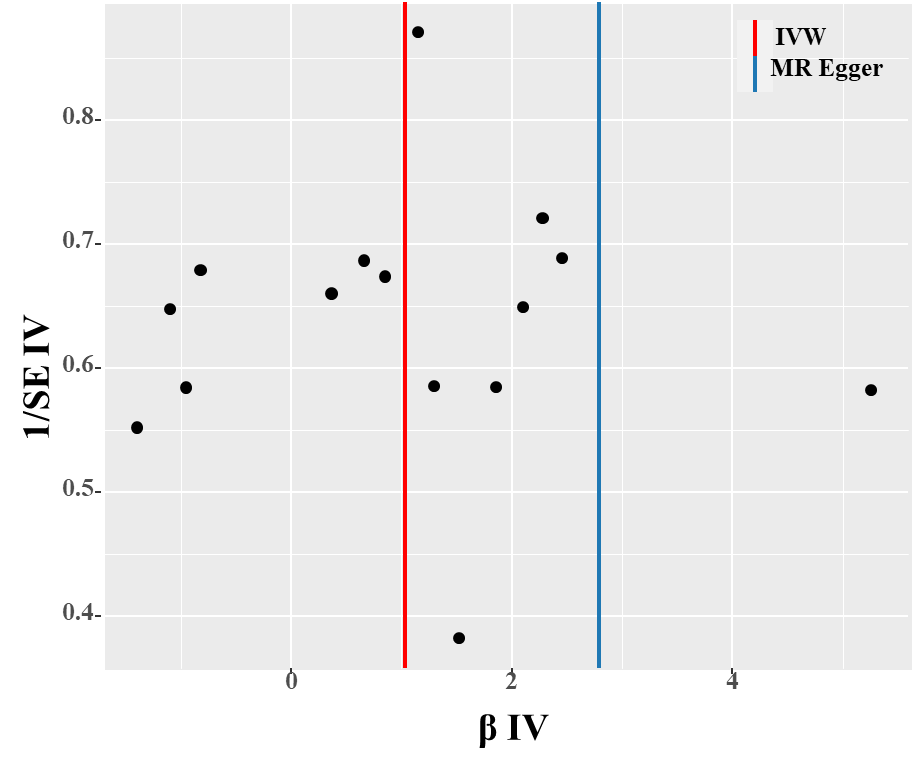


**Figure S4** The funnel plot between genetically predicted snoring and IS in SVMR. IVW, Inverse variance weighted method; SVMR, single-variable mendelian randomization. IV, instrumental variable; IS, ischemic stroke; SE, standard error; MR, mendelian randomization.


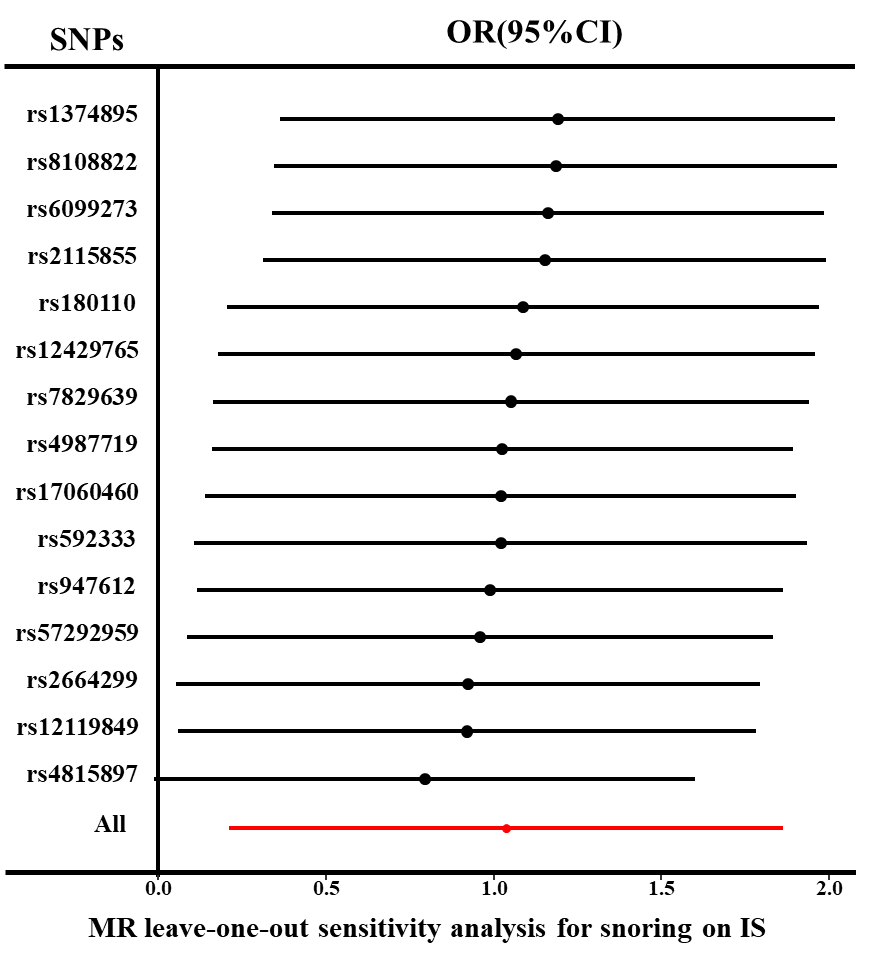


**Figure S5** The leave-one-out sensitivity analyses of snoring and IS. SNP, single nucleotide polymorphism; IS, ischemic stroke; OR, odds ratio; CI, confidence interval; MR, mendelian randomization.


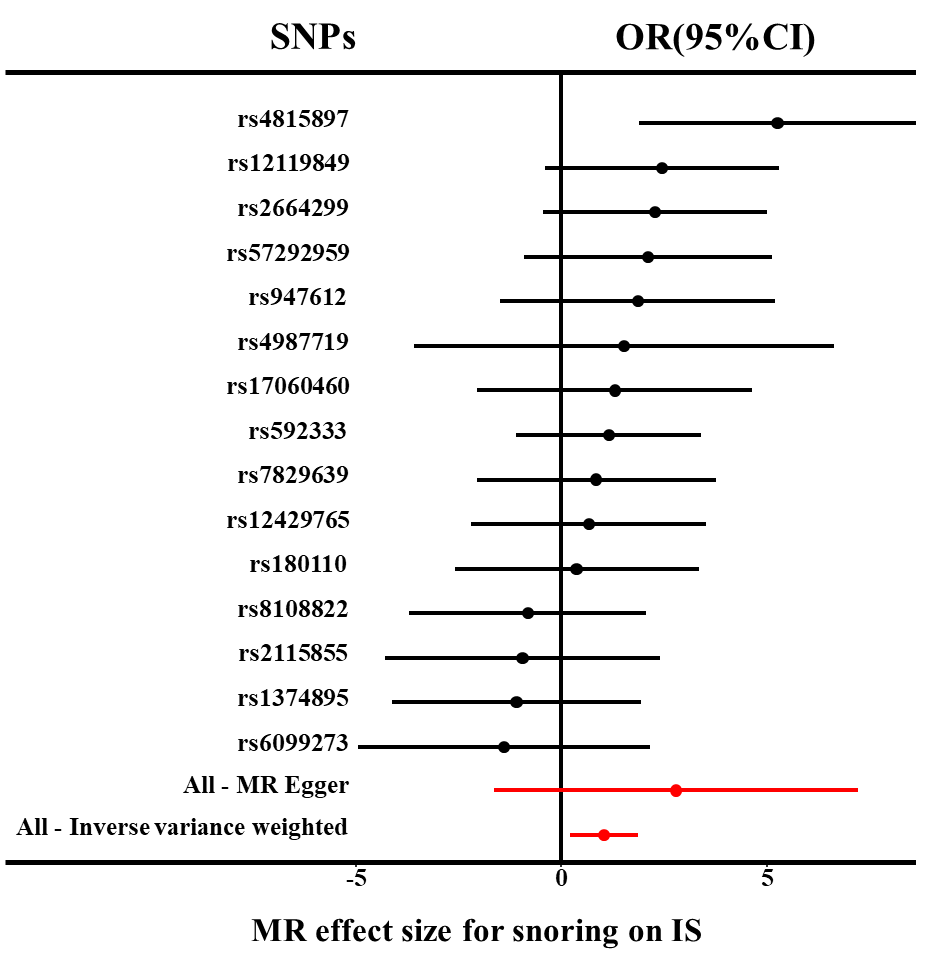


**Figure S6** The results of MR analyses of causal associations between each snoring SNP and IS. SNP, single nucleotide polymorphism; IS, ischemic stroke; OR, odds ratio; CI, confidence interval; MR, mendelian randomization.


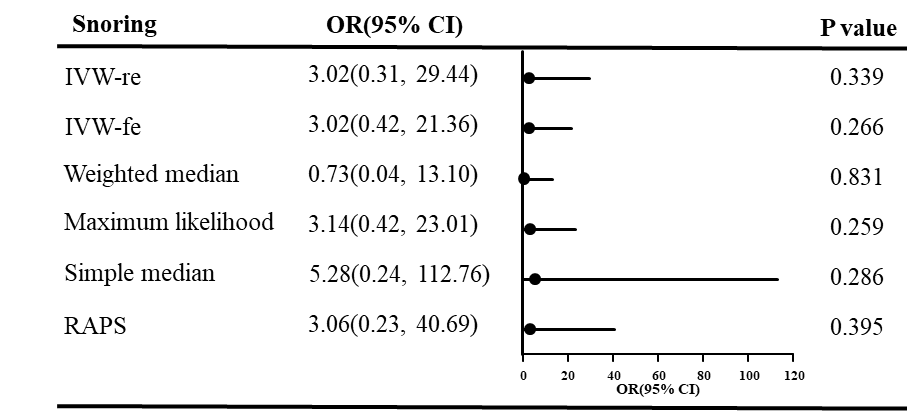


**Figure S7** Causal estimates of snoring on LAS in SVMR. OR, odds ratio; CI, confidence interval; IVW, Inverse variance weighted method; RAPS, robust adjusted profile score; MR, mendelian randomization; LAS, large artery stroke; SVMR, single-variable mendelian randomization.


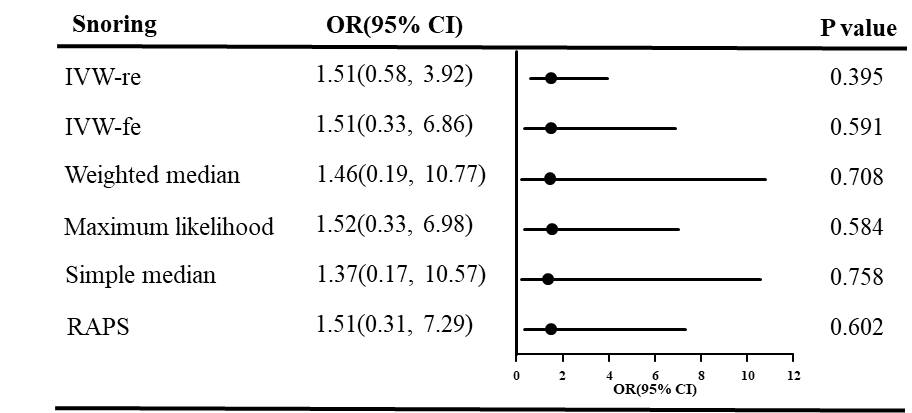


**Figure S8** Causal estimates of snoring on CES in SVMR. OR, odds ratio; CI, confidence interval; IVW, Inverse variance weighted method; RAPS, robust adjusted profile score; MR, mendelian randomization; CES, cardioembolic stroke; SVMR, single-variable mendelian randomization.


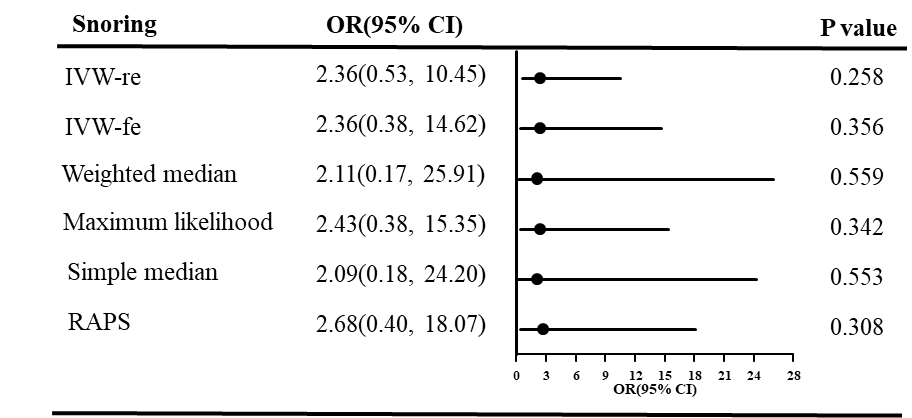


**Figure S9** Causal estimates of snoring on SVS in SVMR. OR, odds ratio; CI, confidence interval; IVW, Inverse variance weighted method; RAPS, robust adjusted profile score; MR, mendelian randomization; SVS, small vessel stroke; SVMR, single-variable mendelian randomization.


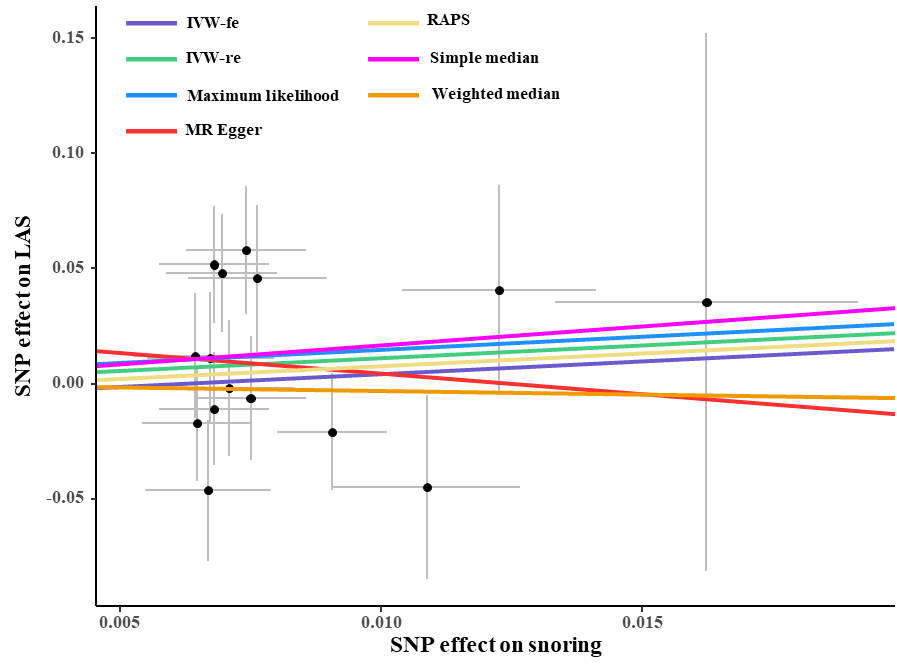


**Figure S10** Scatter plot of the effect size of each SNP on snoring and LAS in SVMR. SNP, single nucleotide polymorphism; LAS, large artery stroke; IVW, Inverse variance weighted method; MR, Mendelian randomization.


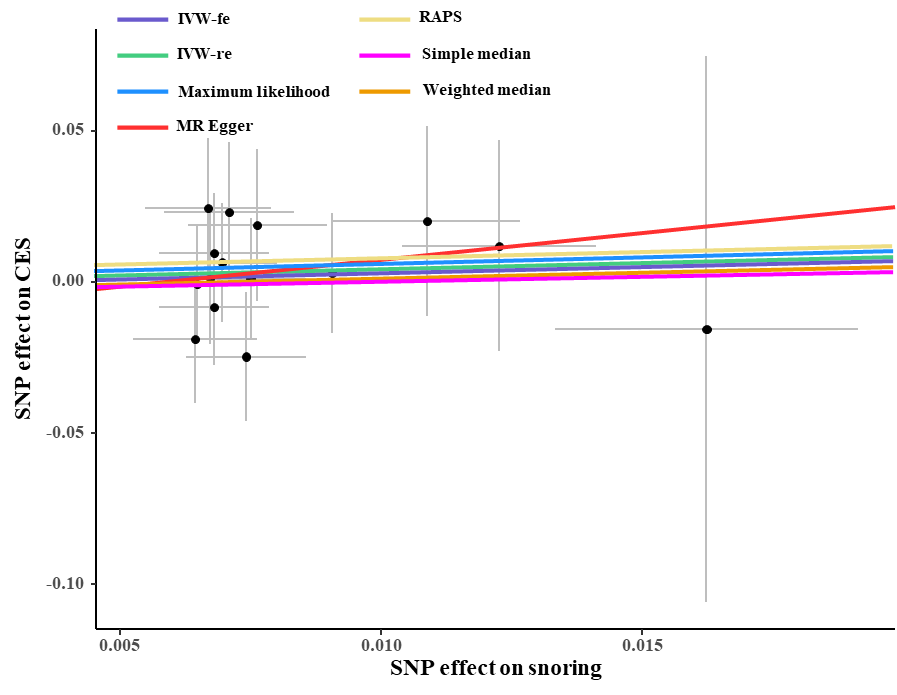


**Figure S11** Scatter plot of the effect size of each SNP on snoring and CES in SVMR. SNP, single nucleotide polymorphism; CES, cardioembolic stroke; IVW, Inverse variance weighted method; MR, Mendelian randomization.


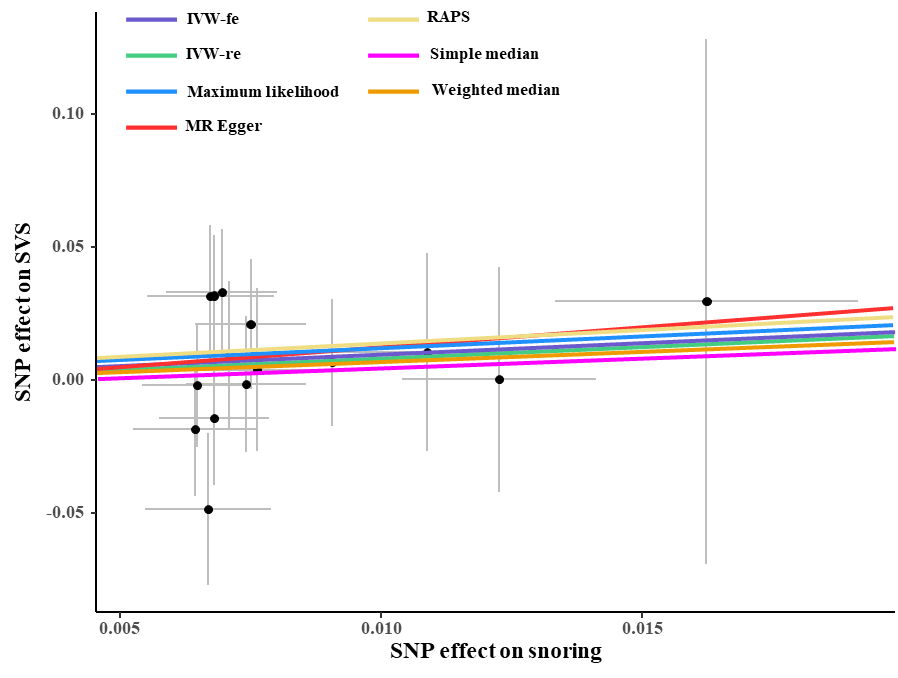


**Figure S12** Scatter plot of the effect size of each SNP on snoring and SVS in SVMR. SNP, single nucleotide polymorphism; SVS, small vessel stroke; IVW, Inverse variance weighted method; MR, Mendelian randomization.


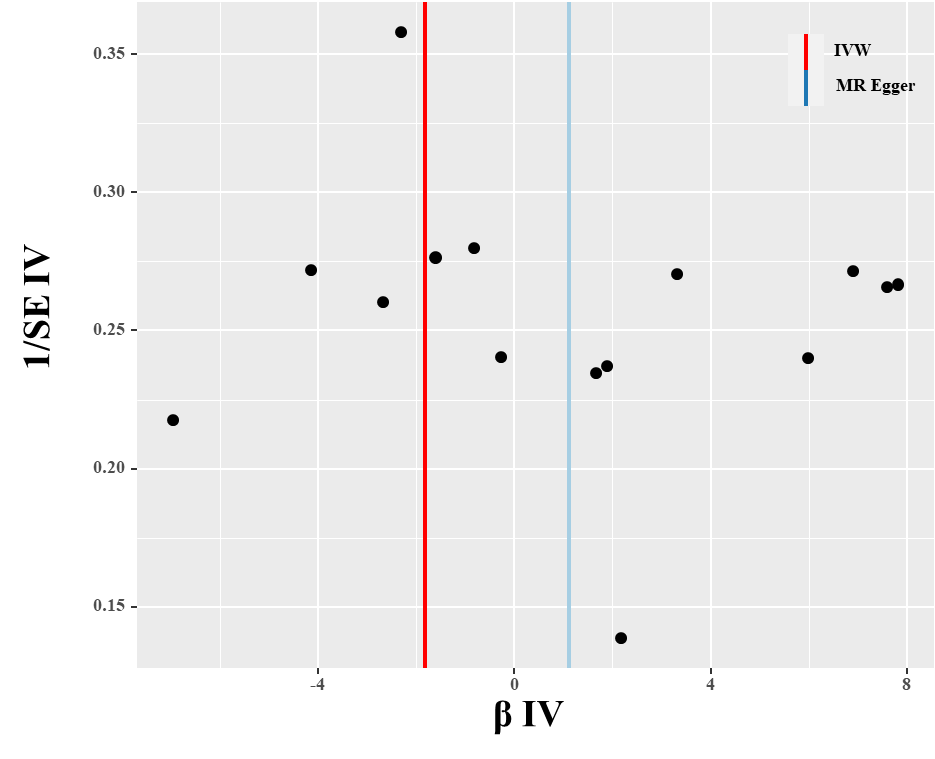


**Figure S13** The funnel plot between genetically predicted snoring and LAS in SVMR. IVW, Inverse variance weighted method; SVMR, single-variable mendelian randomization. IV, instrumental variable; LAS, large artery stroke; SE, standard error; MR, mendelian randomization.


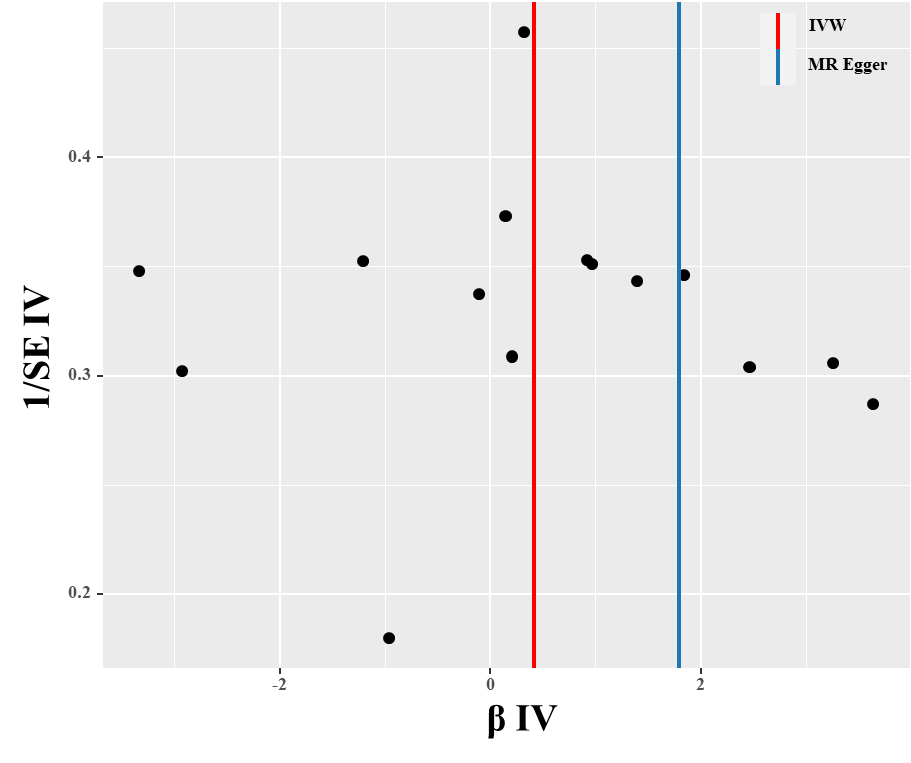


**Figure S14** The funnel plot between genetically predicted snoring and CES in SVMR. IVW, Inverse variance weighted method; SVMR, single-variable mendelian randomization. IV, instrumental variable; CES, cardioembolic stroke; SE, standard error; MR, mendelian randomization.


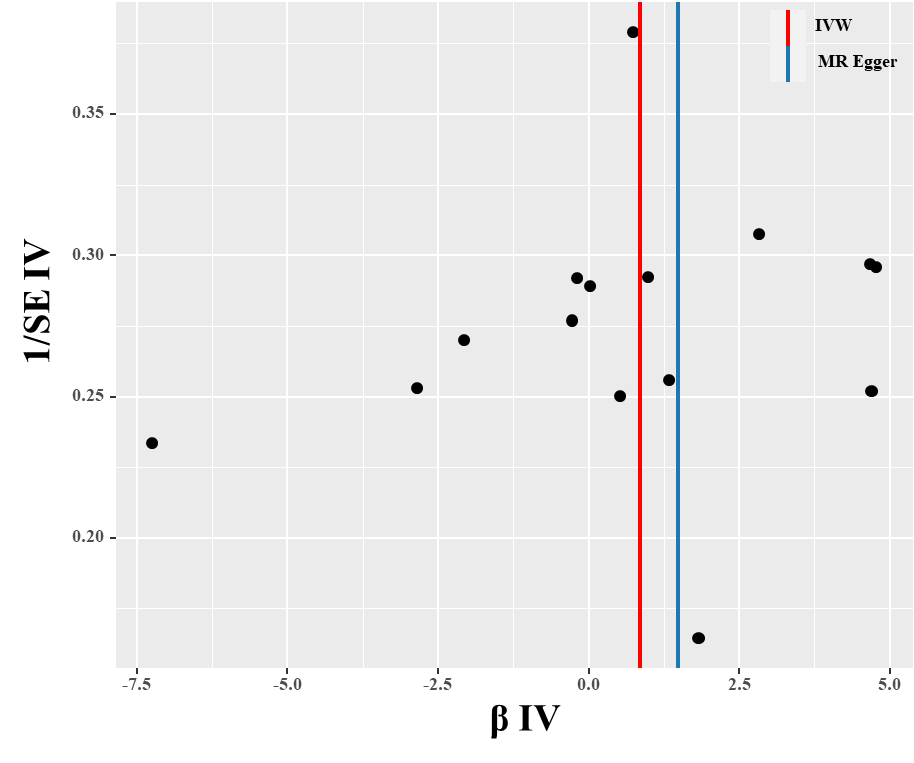


**Figure S15** The funnel plot between genetically predicted snoring and SVS in SVMR. IVW, Inverse variance weighted method; SVMR, single-variable mendelian randomization. IV, instrumental variable; SVS, small vessel stroke; SE, standard error; MR, mendelian randomization.


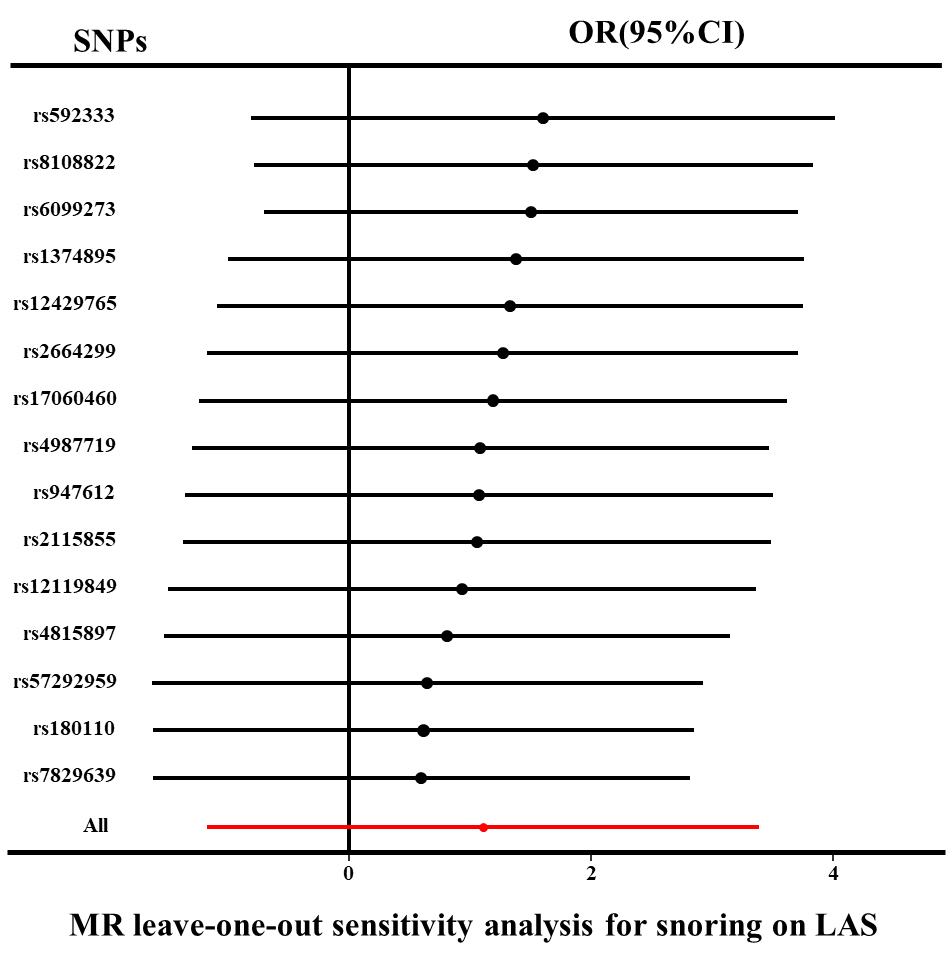


**Figure S16** The leave-one-out sensitivity analyses of snoring and LAS. SNP, single nucleotide polymorphism; LAS, large artery stroke; OR, odds ratio; CI, confidence interval; MR, mendelian randomization.


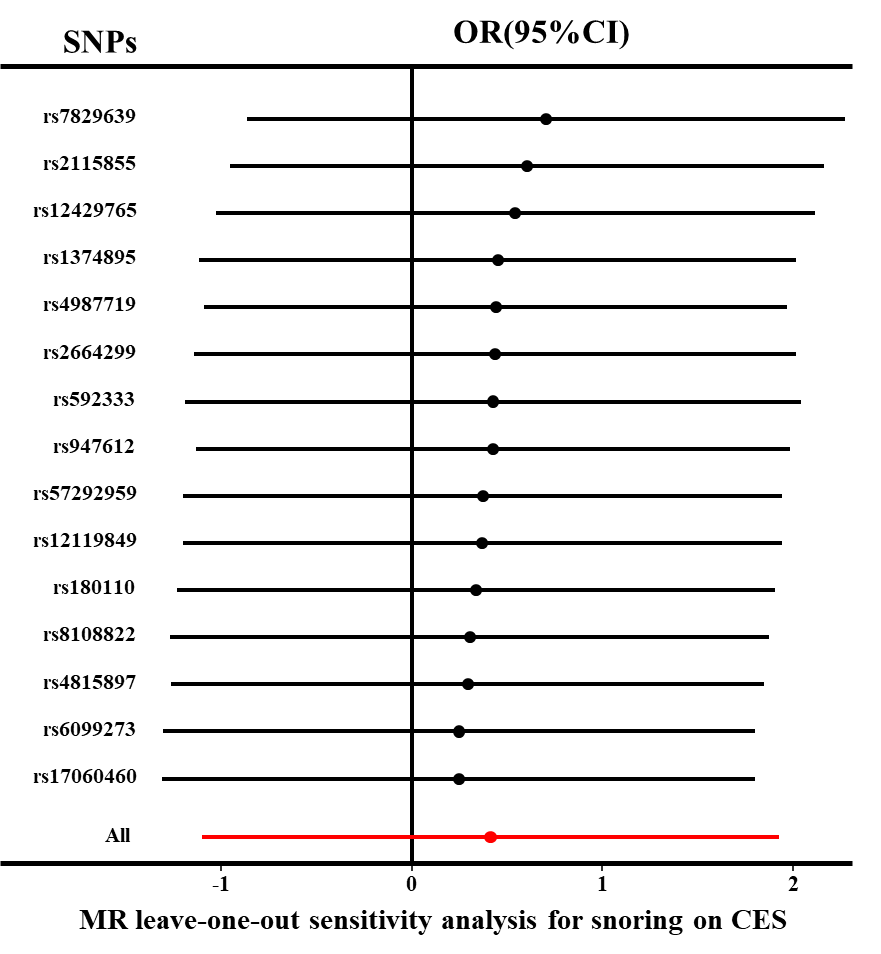


**Figure S17** The leave-one-out sensitivity analyses of snoring and CES. SNP, single nucleotide polymorphism; CES, cardioembolic stroke; OR, odds ratio; CI, confidence interval; MR, mendelian randomization.


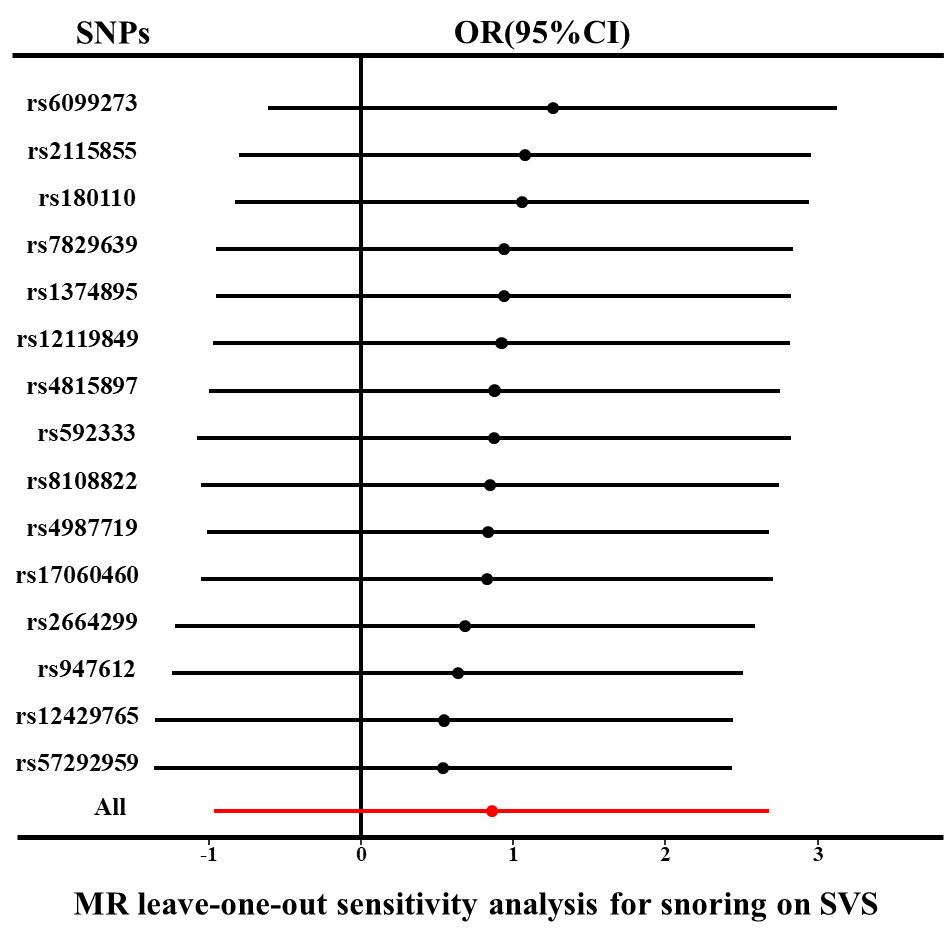


**Figure S18** The leave-one-out sensitivity analyses of snoring and SVS. SNP, single nucleotide polymorphism; SVS, small vessel stroke; OR, odds ratio; CI, confidence interval; MR, mendelian randomization.


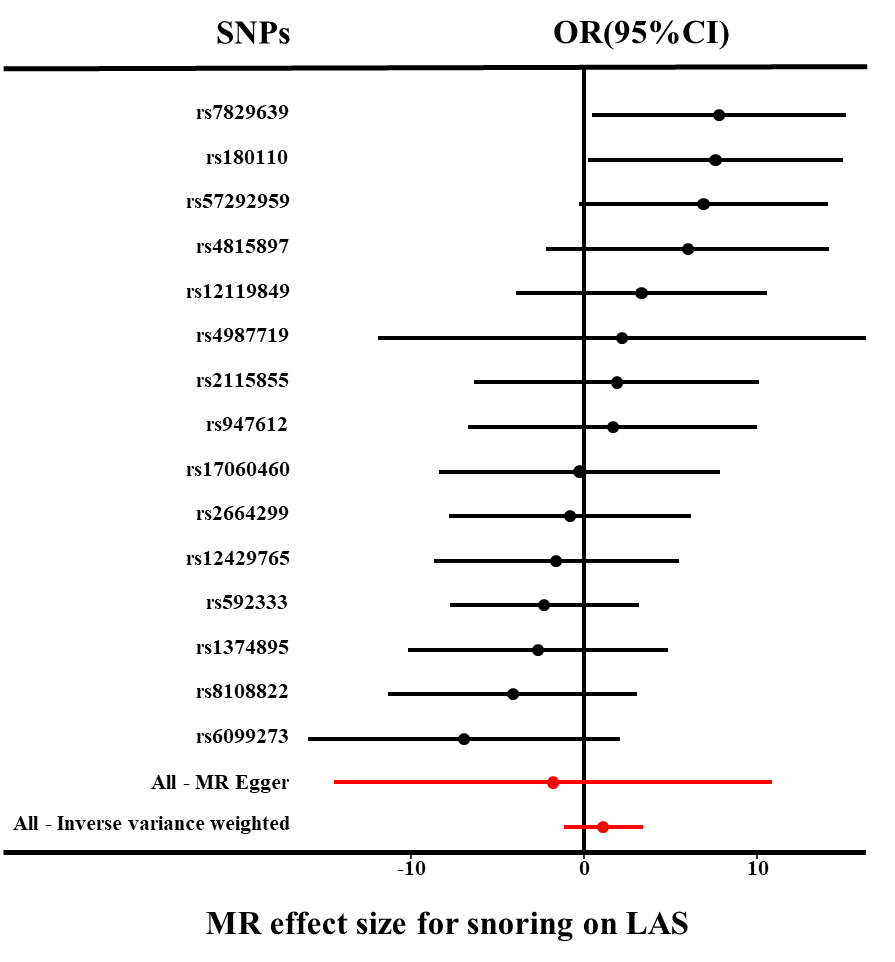


**Figure S19** The results of MR analyses of causal associations between each snoring SNP and LAS. SNP, single nucleotide polymorphism; LAS, large artery stroke; OR, odds ratio; CI, confidence interval; MR, mendelian randomization.


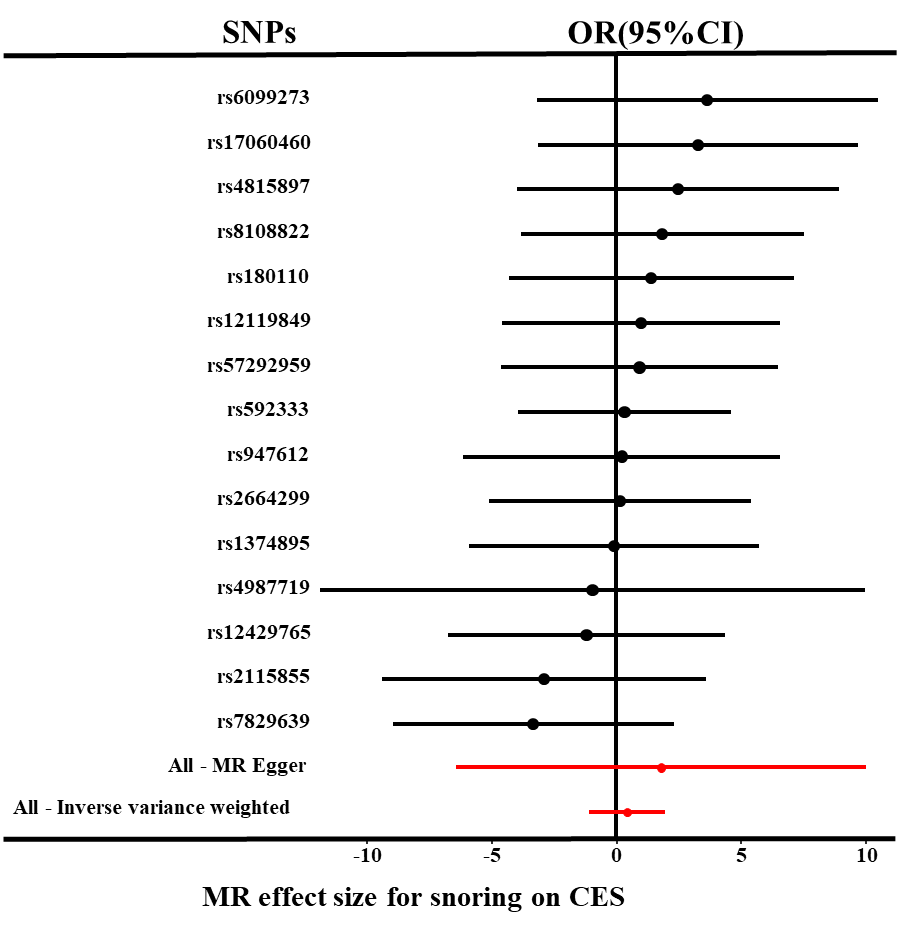


**Figure S20** The results of MR analyses of causal associations between each snoring SNP and CES. SNP, single nucleotide polymorphism; CES, cardioembolic stroke; OR, odds ratio; CI, confidence interval; MR, mendelian randomization.


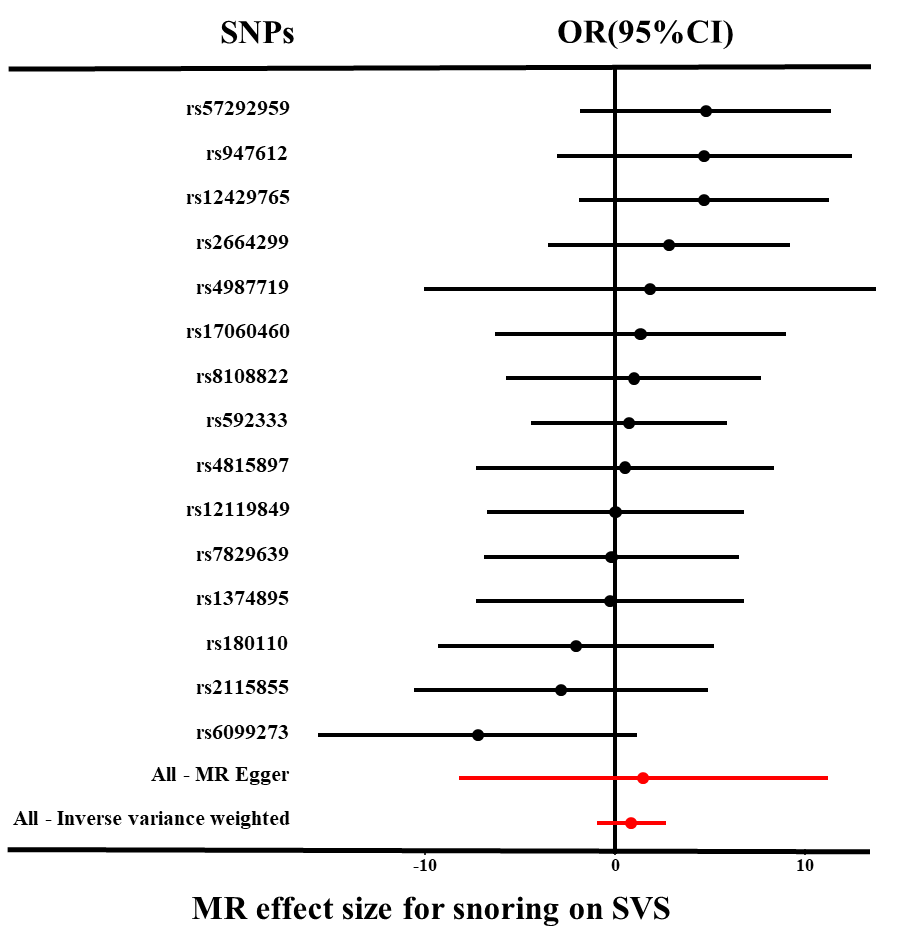


**Figure S21** The results of MR analyses of causal associations between each snoring SNP and SVS. SNP, single nucleotide polymorphism; SVS, small vessel stroke; OR, odds ratio; CI, confidence interval; MR, mendelian randomization.


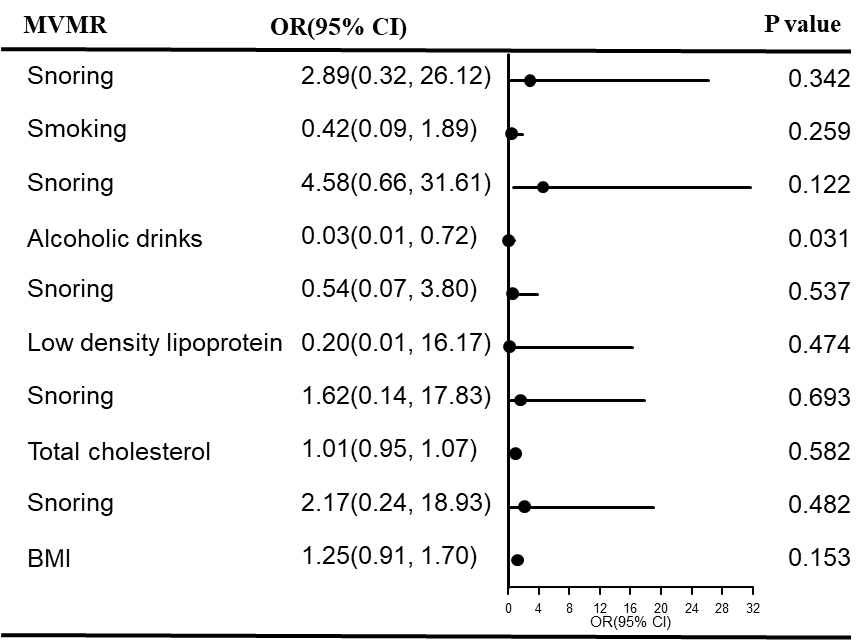


**Figure S22** Causal estimates of snoring on LAS in MVMR. OR, odds ratio; CI, confidence interval; LAS, large artery stroke; MVMR, multivariable mendelian randomization; BMI, body mass index.


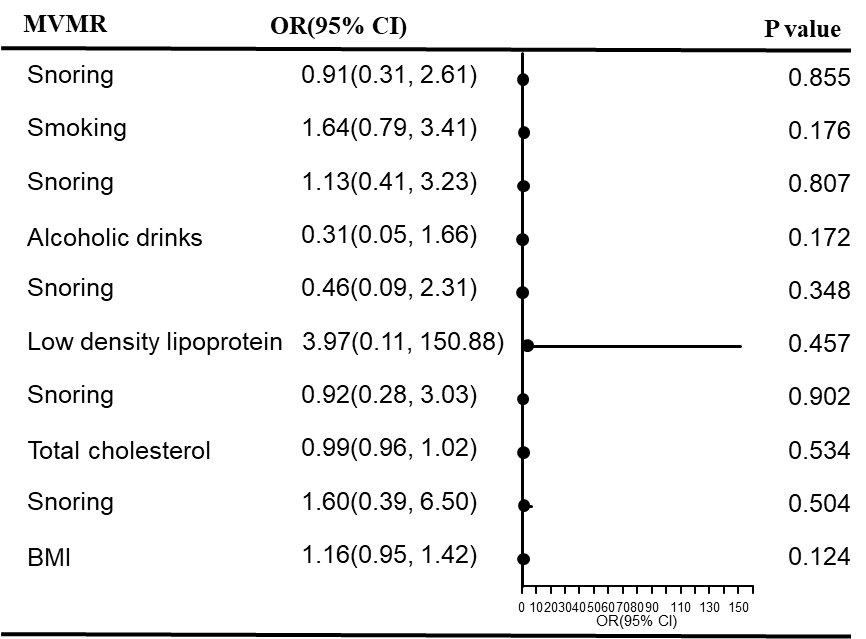


**Figure S23** Causal estimates of snoring on CES in MVMR. OR, odds ratio; CI, confidence interval; CES, cardioembolic stroke; MVMR, multivariable mendelian randomization; BMI, body mass index.


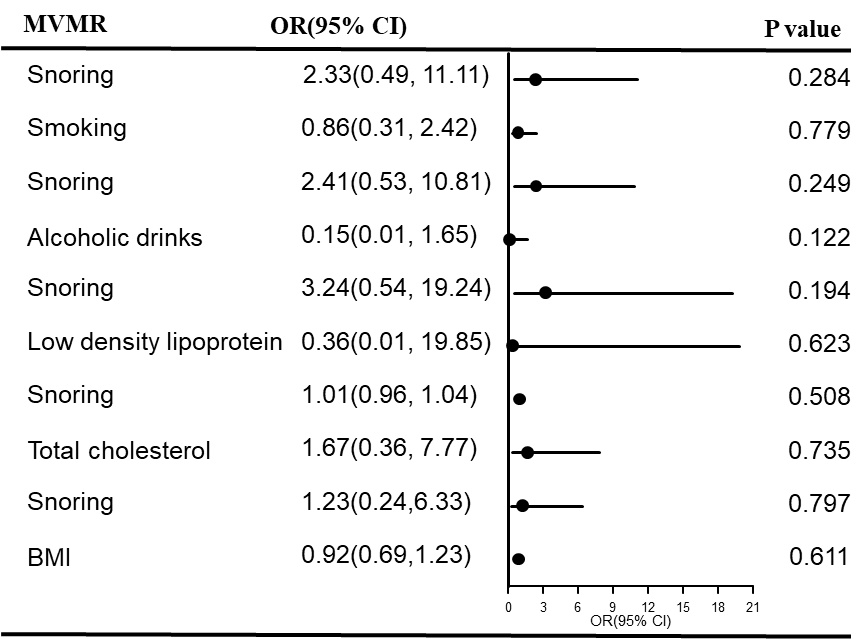


**Figure S24** Causal estimates of snoring on SVS in MVMR. OR, odds ratio; CI, confidence interval; SVS, cardioembolic stroke; MVMR, multivariable mendelian randomization; BMI, body mass index.
